# The Association Between Metabolic Syndrome and the Risk of Endometrial Cancer in Pre- and Post-Menopausal Women: A UK Biobank Study

**DOI:** 10.3390/jcm14030751

**Published:** 2025-01-24

**Authors:** Rebecca Karkia, Gideon Maccarthy, Annette Payne, Emmanouil Karteris, Raha Pazoki, Jayanta Chatterjee

**Affiliations:** 1 College of Health, Medicine and Life Sciences, Brunel University London, Uxbridge UB8 3PH, UK; rebecca.karkia@nhs.net (R.K.); gideon.maccarthy@brunel.ac.uk (G.M.); emmanouil.karteris@brunel.ac.uk (E.K.); raha.pazoki@brunel.ac.uk (R.P.); 2 Academic Department of Gynaecological Oncology, Royal Surrey NHS Foundation Trust, Guildford GU2 7XX, UK; 3 Cardiovascular and Metabolic Research Group, Division of Biomedical Sciences, Department of Life Sciences, College of Health, Medicine and Life Sciences, Brunel University London, Uxbridge UB8 3PH, UK; 4 Department of Information Systems and Computing, Brunel University London, Uxbridge UB8 3PH, UK; annette.payne@brunel.ac.uk; 5 Department of Epidemiology and Biostatistics, School of Public Health, Imperial College London, London SW7 2AZ, UK

**Keywords:** endometrial cancer, UK Biobank, risk stratification, metabolic syndrome, obesity, adipokines

## Abstract

**Background:** Metabolic syndrome (MetS) is a syndrome that comprises central obesity, increased serum triglyceride (TG) levels, decreased serum HDL cholesterol (HDL) levels, raised blood pressure (BP), and impaired glucose regulation, including prediabetic and diabetic glycaemic levels. Recently, the association with endometrial cancer (EC) has been described but it is unclear if the risk associated with MetS is higher than the individual effect of obesity alone. This study investigates the association between MetS components and differing MetS definitions on EC risk and compares the risk of MetS with the risk posed by obesity alone. It also analyses how MetS affects the risk of EC development in the pre- and post-menopausal subgroups. **Methods:** A prospective cohort study was undertaken using data from the UK biobank. Multivariable Cox proportional risk models with the time to diagnosis (years) were used to estimate the hazard ratio (HR) and 95% confidence interval (CI) of MetS and its components on the risk of EC. A subgroup analysis was also undertaken for pre- and post-menopausal participants. Kaplan–Meier (KM) was undertaken to assess the difference in the risk of EC development in differing BMI classes, and in pre- and post-menopausal subgroups. **Results:** A total of 177,005 females from the UK biobank were included in this study. Of those participants who developed EC (*n* = 1454), waist circumference > 80 cm, BMI > 30 kg/m^2^, hypertension > 130/80 mmHg, hyperlipidaemia and diabetes (HbA1C > 48 mmol/L were significant predictors of EC development, with waist circumference being the strongest predictor (HR = 2.21; 95% CI: 1.98–2.47, *p* < 0.001). Comparing the pre- and post-menopausal subgroup, hypertriglyceridaemia and diabetes were the strongest predictors of EC in the pre-menopausal subgroup (HR = 1.53; 95% CI: 1.18–1.99 and HR = 1.51; 95% CI: 1.08–2.12, *p* < 0.05, respectively). Raised waist circumference was not a significant independent predictor in the pre-menopausal subgroup. A KM curve analysis showed a clear distinction between those with and without MetS in the pre-menopausal group, suggesting a benefit of testing for MetS components in pre-menopausal women with obesity. **Conclusions:** Components of MetS, both independently and in combination, significantly increase the risk of EC. Screening those with obesity for MetS in their pre-menopausal years may help to identify those at the highest risk.

## 1. Introduction

Metabolic syndrome (MetS) is a collection of metabolic risk factors for cardiovascular disease and type 2 diabetes mellitus (T2DM). It is a major cause of morbidity and mortality worldwide [1]. There are multiple definitions used, but the one that is common to all is the combination of central obesity, increased serum triglyceride (TG) levels, decreased serum HDL cholesterol (HDL) levels, raised blood pressure (BP), and impaired glucose regulation, including prediabetic and diabetic glycaemic levels [2].

If left untreated, MetS is significantly associated with an increased risk of developing diabetes and its vascular complications, ischaemic heart disease and cerebrovascular disease [3,4]. More recently, however, MetS has been associated with a number of cancers, including colorectal cancer, postmenopausal breast, renal and endometrial cancer (EC) [3,5,6]. EC appears to be one of the cancers most associated with MetS development. In the UK, there are around 10,000 new cases of EC diagnosed annually and up to one-third of these are thought to be preventable by being secondary to obesity [7,8]. In contrast, there are 200,000 new cases of diabetes mellitus (DM) diagnosed annually and latest figures from the National Health Service (NHS) digital suggest that up to 26% of adults in the UK are now obese, with a further 38% falling into the overweight category [9]. Despite this, relatively few who have the top risk factors for EC will go on to develop EC and as such, strategies for risk prediction need to be more nuanced in targeting those at the highest risk [10].

Given the high rates of obesity in the UK and the drive towards precision medicine, it would be beneficial to understand if MetS or its component features can be used to identify those at the highest risk of EC, over and above the risk that body mass index (BMI) adds. We, therefore, sought to investigate the association between EC risk and MetS components, including serum biochemistry variables that pertain to inflammation, insulin resistance and hyperlipidaemia using one of the largest UK-based prospectively collected datasets, the UK Biobank. We compare these predictors to differing BMI levels. A secondary outcome was to conduct a subgroup analysis of pre- and post-menopausal females to determine whether pre-menopausal women with MetS components have a similar risk of EC development as do their post-menopausal counterparts.

## 2. Materials and Methods

### 2.1. Population

**Figure 1:**
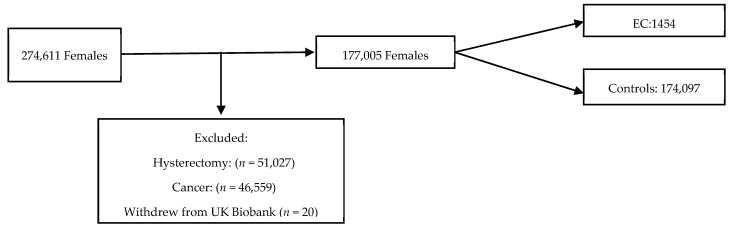
Flow chart of inclusions into study from UK biobank cohort.

The UK Biobank is a large-scale biomedical database containing in-depth information from half a million UK participants aged 39–71 years who were recruited from 22 centres throughout England, Scotland and Wales between 2006 and 2010. The participants were eligible for inclusion if they were female, aged 40–70 years, had not undergone a hysterectomy prior to the study recruitment and had not withdrawn consent for data usage (Figure 1). Women diagnosed with EC prior to or within 12 months of entering the cohort were excluded to reduce the risk of reverse causality. Those who were also diagnosed with a cancer that was not EC were excluded from further study. The participants entered the cohort on the date of their first UK Biobank recruitment appointment. The end of follow-up was defined as 31 December 2020. The data were collected through nurse and self-administered standardised questionnaires and was linked to national cancer and death registries. Censor-time was defined as the period from the date of the first assessment to the occurrence of EC diagnosis, hysterectomy, death, or the end of follow-up, whichever happened first.

### 2.2. Endometrial Cancer

Incident endometrial cancers were identified in the UK Biobank using the International Classification of Diseases (ICD-10) code or from self-reported data.

### 2.3. Metabolic Syndrome Definitions

Metabolic syndrome (MetS) and its components were defined and selected following the World Health Organisation (WHO), International Diabetes Federation (IDF), National Cholesterol Education Programme Adult Treatment Panel III (NCEP:ATPIII) and the Consensus approach standards. Although other definitions exist, these are the most common diagnostic criteria used and hence, the risk associated with these differing definitions was assessed. Central obesity was defined according to waist circumference (≥80 cm in women) [1,11,12,13]. Hypertension was defined as systolic blood pressure (SBP) ≥ 140 (WHO definition) or ≥130 mmHg (IDF, NCEP:ATPIII and Consensus standards definitions) and diastolic blood pressure (DBP)  ≥ 90 (WHO definition) or ≥85 mmHg (IDF, NCEP:ATPIII and Consensus standards), or previously diagnosed or undergoing treatment for hypertension. Elevated triglycerides were defined as a plasma triglyceride level  ≥ 1.7 mmol/L (150 mg/dL) or a prior diagnosis of elevated triglycerides or ongoing use of triglyceride lowering medication. Reduced HDL was defined as plasma HDL  < 1.29 mmol/L (50 mg/dL) or being treated with lipid-altering medication. Hyperglycaemia was defined as fasting blood glucose ≥ 5.6 mmol/L (100 mg/dL) or a prior diagnosis of type 2 diabetes or medical treatment for type 2 diabetes. The above five conditions are the MetS components. Different definitions were used to diagnose MetS (Table 1).

### 2.4. BMI Definitions

BMI is not a MetS predictor apart from in the WHO 1999 criteria, where BMI ≥ 30 kg/m^2^ is a feature; however, BMI is a frequently recorded piece of anthropometric data in primary and secondary care settings. For this reason, BMI was used as a comparator in the statistical analysis to compare the differences between MetS predictors and BMI on the risk of EC development. The WHO classification for BMI was used [14]. Normal weight was used as the reference and was defined as a BMI ≤ 24.9 kg/m^2^. Overweight was defined as a BMI of 25.0–29.9 kg/m^2^. Class I obesity was defined as a BMI of 30.0–34.9 kg/m^2^. Class II obesity was defined as a BMI of 35.0–39.9 kg/m^2^ and Class III obesity was defined as a BMI ≥ 40.0 kg/m^2^.

### 2.5. Statistical Analysis

In the baseline characteristic description, categorical variables were expressed using percentages and frequencies, while continuous variables were presented using means (standard deviation, SD) for normally distributed variables, and medians (interquartile range) for skewed variables. Cox proportional risk models with the time to diagnosis (years) was used to estimate the hazard ratio (HR) and 95% confidence interval (CI) of MetS and its components on the risk of EC. The proportional risk hypothesis was tested using the Schoenfeld residual method. All models were adjusted for age, menopausal status, use of contraception, use of hormone replacement therapy, nulliparity and smoking, as they are known confounding factors in endometrial cancer and remained significant in this analysis. Multicollinearity was assessed with the variance inflation factor (VIF). Cumulative risk was assessed using Kaplan–Meier (KM) curves and log-rank tests. KM curves were generated to illustrate the impact of individual MetS components (Consensus criteria), differing MetS definitions, the effect of different WHO BMI classes, the combination of obesity and diagnosed MetS (Consensus criteria) and lastly, BMI class, MetS status and menopause status on the risk of EC with significant differences between groups (log-rank test, *p* < 0.001). The models were adjusted for age, menopausal status, contraception, hormone replacement therapy, parity and smoking.

Statistical analyses were carried out using R software (version 3.5.0, R Foundation for Statistical Computing, Vienna, Austria) and STATA (version 16.1, StataCorp LLC., College Station, TX, USA). All statistical tests were two-tailed, with *p* < 0.05 considered statistically significant.

### 2.6. Ethics

The study was approved by the Northwest Multi-Centre Research Ethics Committee (16/NW/0274), the Patient Information Advisory Group (England and Wales) and the Community Health Index Advisory Group (Scotland). All participants provided written informed consent and were free to withdraw from study inclusion. The study was conducted in accordance with the Declaration of Helsinki.

## 3. Results

A total of 1454 females were diagnosed with EC over a median follow-up time of 6.4 years. The baseline characteristics of both cohorts are displayed in Table 2. Table 3 summarises the frequency of MetS characteristics in both cohorts, with Supplementary Figures S1 and S2 displaying this graphically in frequency histograms. Significant differences were observed between the control and EC groups in several baseline characteristics. The ethnicity in both groups was predominantly white (92.4% in controls vs. 93.1% in EC). The EC group had a higher median index of deprivation (14.0 vs. 12.9, *p* < 0.001). The EC group was older at recruitment, with a median age of 60.0 years, compared to 55.0 years in the controls (*p* < 0.0001). The height was slightly lower in the EC group (162.0 cm vs. 162.6 cm, *p* = 0.0002), and the EC group had a significantly higher weight (76.7 kg vs. 68.5 kg) and BMI (29.4 kg/m^2^ vs. 25.8 kg/m^2^) (*p* < 0.0001 for both). Waist circumference was also greater in the EC group (91 cm vs. 82 cm, *p* < 0.0001). The age at menarche did not differ significantly, but menopausal status did, with a larger proportion of women in the EC group being post-menopausal (81.9% vs. 66.4%, *p* < 0.0001). The age at menopause was also slightly higher in the EC group (52.0 years vs. 51.0 years, *p* < 0.0001). The EC group had a lower proportion of oral contraceptive pill users (70.4% vs. 81.9%, *p* < 0.0001), but a higher proportion used hormone replacement therapy (36.1% vs. 29.0%, *p* < 0.0001). Smoking habits differed, with more never smokers in the EC group (66.5% vs. 60.9%) and fewer current smokers (4.7% vs. 8.6%, *p* < 0.0001). The prevalence of diabetes was higher in the EC group (8.1% vs. 3.3%, *p* < 0.0001). Additionally, a higher proportion of the EC group were taking cholesterol-lowering medications (18.7% vs. 10.3%) and antihypertensives (15.1% vs. 8.9%) (*p* < 0.0001 for both). A small but significant difference in polycystic ovarian syndrome (PCOS) was observed, with more cases in the EC group (0.3% vs. 0.2%, *p* < 0.001). When examining the frequency with which the cohorts met the diagnostic criteria for MetS, the Consensus definition classified the highest proportion of cases and controls as MetS, as compared to the WHO definition with the least. In total, 39% of the EC group and 22% of the control group met the Consensus diagnostic criteria for MetS, whereas only 11% and 5% of the EC group and control group met the WHO criteria.

**Figure 2:**
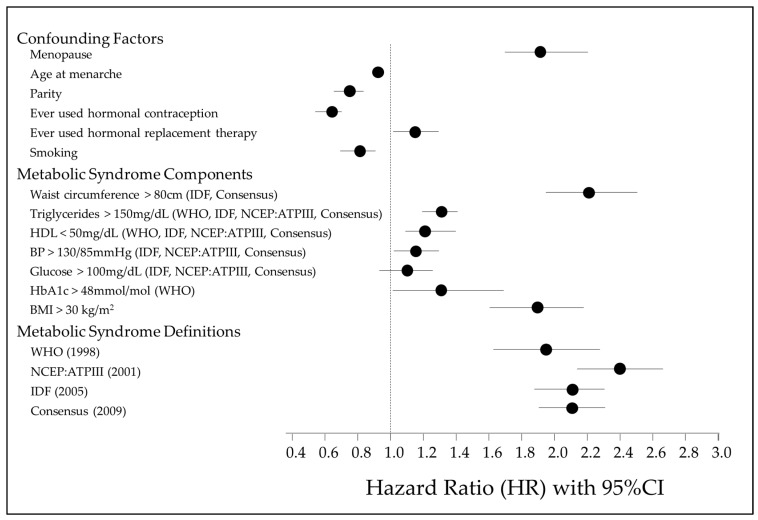
Forest plot illustrating association of MetS individual components, MetS definitions, confounding factors and risk of EC development [1,12,13,14].

The results of a multivariable Cox proportional hazards regression examining the associations between MetS, its various components, and risk of EC are shown in Figure 2 and Supplementary Table S1. Figure 1 illustrates the factors controlled for in the multivariable Cox regression, the MetS individual components and the four diagnostic definitions of MetS. A waist circumference > 80 cm is the strongest MetS risk factor associated with EC development (HR 2.21, 95% CI 1.98–2.47, *p* < 0.001). All MetS components, apart from fasting glucose (>100 mg/dL), are significant independent risk factors. However, regardless of the definition used, a diagnosis of MetS has a stronger association with EC development than individual factors alone.

The age at menarche, nulliparity, ever use of HRT, BMI > 30 kg/m^2^, waist circumference > 80 cm, triglyceride levels > 150 mg/dL, HDL levels < 50 mg/dL and HbA1c levels > 48 mmol/mol were all significant independent risk factors for EC development. The ever use of the oral contraceptive pill (OCP), previous or current history of smoking, and higher age at menarche were significant risk-reducing features. Glucose > 100 mg/dL was not a significant independent predictor of EC risk but a raised HbA1c over 48 mol/mol was.

Analysing the different diagnostic criteria for MetS, the overall risk of EC development was assessed. All the diagnostic criteria for MetS that were analysed showed significant positive associations with EC risk and did not differ majorly. The strongest association was observed in the cohort meeting the diagnostic criteria for MetS by the NCEP:ATPIII definition (HR = 2.39, 95% CI 2.14–2.66, *p* < 0.001). The WHO diagnostic criteria had the weakest association with EC risk (HR = 1.93, 95% CI 1.63–2.28, *p* < 0.001) (Table 4). Of those who developed EC, the shortest time to EC development was seen in those who met the NCEP definition, as compared to any other definition (Supplementary Figure S3).

**Figure 3:**
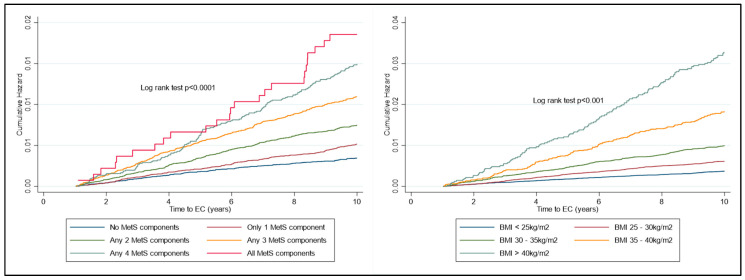
KM plot to represent cumulative risk associated with MetS components and with BMI WHO Group. Risk is seen to increase significantly with both the number of MetS components diagnosed with and also amongst the increasing classes of obesity.

The risk of developing EC increased significantly over time, depending on the number of components of metabolic syndrome present (Figure 2). The risk of developing EC increased significantly over time, depending on the BMI category, with the key inflexion point being in those with BMI ≥ 35 kg/m^2^ (Figure 3).

**Figure 4:**
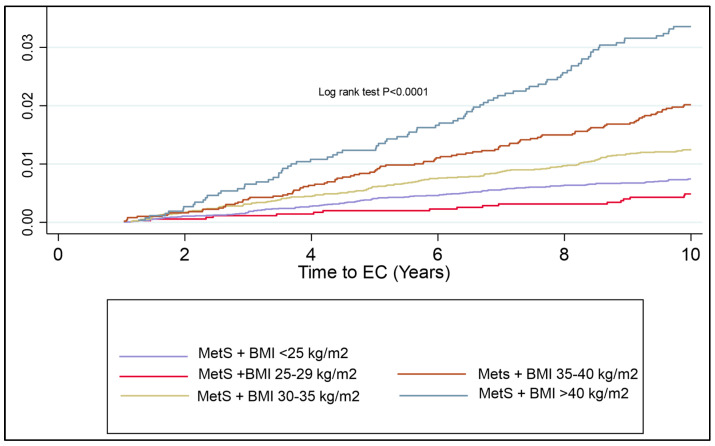
KM plot to represent cumulative risk associated with BMI and MetS components combined. Risk of EC development is seen to significantly increase in all classes of obesity, with the most apparent inflexion point being patients with Class II obesity or more.

Despite this not being a component of MetS per se, given the strength of the association of BMI with EC risk, a KM plot was generated to assess the additional risk of BMI alone with a diagnosis of MetS. Morbid obesity, regardless of whether there is concurrent MetS, has a similar risk of EC development over time. The risk of EC development over time is markedly different in each BMI group. There was a significantly shorter time to EC development in the cohort with MetS and Class I, II and III obesities as compared to the cohort with MetS in the normal weight and overweight group (log rank test, *p* < 0.0001) (Figure 4).

**Figure 5:**
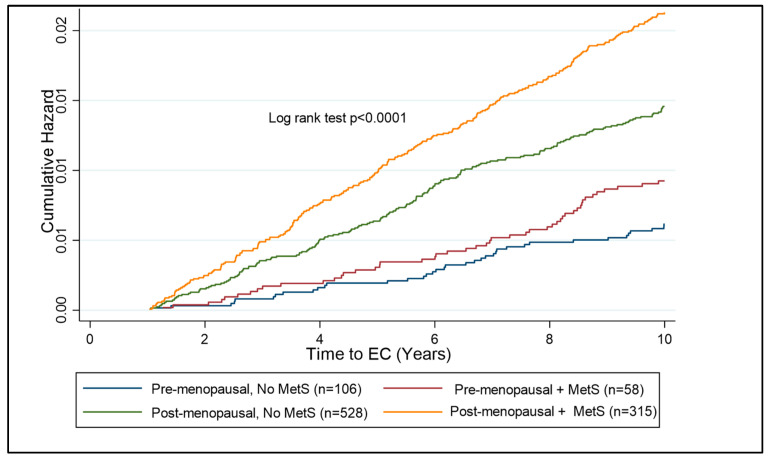
Kaplan–Meier curve showing cumulative hazard of EC associated with MetS in pre- and post-menopausal women with obesity (BMI ≥ 30 kg/m^2^). Post-menopausal women with MetS are at the highest risk of EC development as compared to other subgroups. The risk over ten years is significantly lower in the pre-menopausal subgroup than the post-menopausal subgroup; however, there is a clear distinction between those with and without MetS in the pre-menopausal group, suggesting a benefit of testing for components of MetS in pre-menopausal women with obesity.

A subgroup analysis of pre- and post-menopausal females was undertaken to establish the risk of MetS components on the future risk of EC. The post-menopausal subgroup was the larger subgroup, with 1201 participants subsequently diagnosed with EC and 118,406 females forming the control cohort. The pre-menopausal subgroup comprised 249 participants who developed EC and 55,299 controls (Table 5). Looking at the components of MetS in pre-menopausal females, hypertriglyceridemia and HbA1c > 48 mmol/mol were the strongest independent predictors of EC risk (HR = 1.53; 95% CI 1.18–1.99, *p* < 0.001 and HR = 1.51; 95% CI 1.51; 95% CI 1.08–2.12, *p* = 0.017, respectively). In post-menopausal females, waist circumference, hypertriglyceridaemia, HDL < 50 mg/dL, hypertension, and raised HbA1c were all independent predictors of EC risk. Pre- and post-menopausal women with BMI > 30 kg/m^2^ were assessed to identify the additional risk that MetS has, in association with EC development. In the pre-menopausal years, there is a significant additional risk of having MetS over and above that of having a high BMI alone (Figure 5).

An analysis of multicollinearity with the variance inflation factor (VIF) showed no significant multicollinearity amongst the MetS predictors (R^2^ ranging from 0.009 to 0.016 with VIF ranging between 1.01 and 1.07).

## 4. Discussion

The principal findings of the study show that increased waist and hip circumference, increased arterial blood pressure, abnormal lipid profile and deranged glucose metabolism are all associated with an increased risk of developing EC, regardless of whether they are identified in pre-menopause or post-menopause. Furthermore, all components of MetS are significant independent predictors of EC development. This supports the hypothesis that MetS is strongly associated with EC development and thus, in effect, may be used as a predictor of EC, even in the pre-menopausal period. Although not a feature of the contemporary MetS definitions, BMI is the strongest known independent risk factor for EC development and the most commonly reported anthropometric measure in the primary care setting. Given this, comparing the risk of EC development in association with MetS predictors or MetS diagnosis, as compared to BMI alone, showcases the utility of making this differentiation.

There have been multiple studies demonstrating the associations of MetS with cancer. A recent 2022 analysis examined the relationship between MetS and the risk of thirteen IARC obesity-associated cancers [6]. It pooled the results of 63 studies of the cancer risk in adults (all age groups > 18 years age) without MetS versus with MetS. The effect estimates for the risk of cancer (adjusted for alcohol consumption or cigarette smoking in 68% of studies) were as follows: 1.13–6.73 for breast; 1.14–2.61 for colorectal; 1.18–2.50 for gastric; 1.59–2.13 for pancreas; 2.13–5.06 for hepatocellular carcinoma and 1.37–2.20 for endometrial cancer [6]. This is very much similar to the risk demonstrated by MetS in this UK Biobank study. Few studies to date have directly compared the differing MetS criteria and their association with EC. In this cohort, the overall risk associated with the different definitions did not differ markedly (HR range 1.9–2.4); however, the size of the cohorts diagnosed as having MetS did, with the WHO criteria diagnosing the fewest and the Consensus criteria diagnosing over one-fifth of the control cohort and nearly forty percent of the EC group. The NCEP criteria was associated with the highest risk of EC development (HR = 2.39, 95% CI 2.14–2.66, *p* < 0.001). Given the similarity of the other criteria, this increased risk is likely an effect of the higher waist circumference threshold of >88 cm, which further differentiates those with central adiposity.

Arguably, adiposity is the largest hallmark of MetS as it is linked to several metabolic abnormalities, including insulin resistance and inflammation, that are associated with EC development. There is evidence that excess visceral adipose tissue, in particular, is associated with adverse metabolic, dyslipidemic, and atherogenic obesity, as compared to subcutaneous fat. Central adiposity is typically assessed by waist circumference or the waist-to-hip ratio; however, there is poor agreement in the literature about the single best anthropometric measure for the assessment of EC risk. In the UK Biobank cohort, a waist circumference > 88 cm was the strongest predictor and BMI > 30 kg/m^2^ (WHO criteria) was the weakest. In the California Teachers’ Study, waist circumference and waist-to-hip ratio were positively associated with EC risk after the adjustment for BMI [15]. In contrast, the Nurses’ Health Study did not report an independent association with these measures, while the European Prospective Investigation into Cancer and Nutrition cohort (EPIC) reported an independent association with waist circumference but not waist-to-hip ratio [16,17]. In the E2C2 study, the overall pooled estimate for obesity and EC risk was 2.65 (95% CI: 2.43–2.90) in those with a BMI of 30–35 kg/m^2^ and 4.66 (95% CI: 3.78–5.75) in individuals with Class II obesity (BMI > 35 kg/m^2^), which is similar to the findings in this cohort study [18,19].Whilst the risk of having a BMI > 40 kg/m^2^ was the strongest assessed independent predictor in this study (HR = 5.87; 95% CI: 4.53–7.62), this reflects only 11% of the EC subgroup and 2.4% of this UK Biobank cohort overall. Furthermore, in one study assessing a cohort of bariatric patients with BMI > 40 kg/m^2^ of the 72 women assessed, 10 (14%) had an occult endometrial abnormality at baseline, 4 with frank EC and 6 with atypical hyperplasia [20]. Of the ten women with endometrial pathology, eight were pre-menopausal and eight had known or undiagnosed diabetes or insulin resistance. The risk of this subgroup of obese women is thus well understood and the difficulty, perhaps, lies in stratifying the risk of those falling into the lower classes of obesity.

Interestingly, in the pre-menopausal subgroup analysis, diabetes (HbA1C > 48 mmol/mol) and hypertriglyceridemia was associated with a higher risk of EC than central obesity or BMI > 30 kg/m^2^, which was not the case in the post-menopausal subgroup. The combination of obesity and MetS diagnostic components in the pre-menopausal subgroup is, therefore, a potentially key clinical differentiator.

Insulin resistance and hyperinsulinemia are associated with EC risk independent of obesity [21,22]. Insulin may act directly on endometrial tissue as a mitogenic and antiapoptotic growth factor [21,22,23,24]. Insulin can also increase IGF-I bioactivity and increase the bioavailability of free oestrogens and androgens through the downregulation of SHBG and upregulation of ovarian sex steroid production [21,25]. Amongst the UK Biobank cohort, fasting glucose ≥ 100 mg/dL was not a significant independent predictor of EC after multivariable regression; however, HbA1c > 48 mmol/L was. These findings are in keeping with a population-based prospective cohort study where women with type 2 diabetes had a 2-fold higher risk of EC development [26]. A potential explanation for the discrepancy in findings is that glycaemic treatment, such as metformin, may modify risk. Whilst in vitro studies have been promising, larger trials and cohort studies have failed to find a significant benefit. A recent Cochrane review, which included only two small randomised controlled trials, did not find sufficient evidence to confirm whether metformin lowers the risk of EC development [27]. More recently, randomised trials, such as the feMME trial, have also found no significant benefit [28].

Whilst the evidence for metformin for the reduction in future EC risk is uncertain, there is good evidence to suggest that primary prevention strategies are effective. Weight loss or bariatric surgery is associated with a significant reduction in cancer risk. One recent meta-analysis on the evidence for the prevention of future cancers following bariatric surgery examined eight studies pertaining to EC, with over 346,430 women in the bariatric surgery group and 1,075,024 women in the control group. Bariatric surgery was linked to a significant decrease in EC incidence (RR 0.38, 95% CI 0.26–0.55, *p* < 0.00001). There was also a significant reduction in the risk of hepatocellular carcinoma (RR 0.35, 95% CI 0.22–0.55, *p* < 0.00001), colorectal cancer (RR 0.63, CI 0.50–0.81, *p* = 0.0002), pancreatic cancer (RR 0.52, 95% CI 0.29–0.93, *p* = 0.03), breast cancer (RR 0.56, 95% CI 0.44–0.71, *p* < 0.00001) and ovarian cancer (RR 0.45, 95% CI 0.31–0.64, *p* < 0.0001) [29]. A similar meta-analysis examining the effect of weight loss and weight loss associated with bariatric surgery in EC risk reduction suggested similar results, yielding a 59% lower risk of EC following bariatric surgery (OR 0.41, 95% CI 0.22 to 0.74) and an estimated risk reduction of 5–40% with conventional weight loss [30]. Furthermore, there appears to be growing evidence that glucagon-like peptide 1 receptor agonists are associated with a reduced risk of obesity-related cancers, with particular evidence where diabetes and obesity are concurrent. The same study reported a 36% risk reduction in EC development (HR, 0.74; 95% CI, 0.60–0.91) [31]. The benefits of progestin therapy for inducing regression in endometrial hyperplasia and pre-cancerous endometrial changes are also well established and thus, evidence-based risk-reduction measures could suitably be used for those at the highest risk [32].

One of the major strengths of this study is its large size and the fact that it is the only study using the UK Biobank to focus on the association between MetS and EC individually. The prospective design minimises the selection bias arising from the inappropriate selection of control subjects. All cases were incident cases diagnosed at least one year after recruitment, reducing the risk of reverse causality. The risk factors identified in this study remained true risk factors regardless of menopausal status, offering an opportunity for risk reduction strategies prior to the average age of onset of the disease in the seventh decade.

One of the limitations of the UK Biobank is the relative short duration of follow-up. This is especially so seeing as the average age of EC development is in the six or seventh decade and the age at recruitment was 60 and 55 for the cases and controls, respectively. Another limitation was that the exposures, along with the important covariates, were measured only once at cohort entry, so the potential changes over time were not accounted for. A further limitation of the study was the inability to determine the association of PCOS and EC risk. As in the study of Hutt et al. we know, from meta-analysis, that PCOS is a major contributing risk factor to EC development and is closely interlinked with insulin resistance [10]. Unfortunately, in the UK Biobank, very few patients were reported as having had a diagnosis of PCOS. It is likely that this is due to their age at the time of the UK Biobank study and the fact that PCOS is usually diagnosed in early reproductive life. Finally, and perhaps most importantly, is the fact that the UK Biobank population although large, is relatively homogenous, with the majority of patients being of Caucasian white origin. This limits the generalisability of results to the UK population. However, similar findings have been demonstrated in European- and United States-based cohorts, showing similar findings amongst more ethnically diverse groups [15,16,17,18,19].

## 5. Conclusions

This study supports the hypothesis that MetS and its individual components raised waist circumference, hypertension, hyperlipidemia, and diabetes, significantly increasing the risk of EC development and thus, may be used as clinical risk-predictors. In the pre-menopausal period, diabetes and hyperlipidaemia are stronger predictors than BMI or waist circumference. Despite published data showing strong associations between components of MetS and EC risk, there are currently no nationally recommended screening strategies and risk reduction programmes in place despite the evidence for certain measures, such as bariatric surgery. Using the Consensus diagnostic criteria, MetS was diagnosable in up to 22% of the control cohort, and, strikingly, in 39% of patients who went on to develop EC. This is likely due to the large overlap between the two conditions and their pathogenesis. Screening the females with obesity for other components of MetS may stratify those at the highest risk of EC over and above the risk posed by obesity alone, especially in the pre-menopausal period. Furthermore, mediating the conditions associated with MetS may modify the risk of EC, as well as, potentially, a number of other obesity driven cancers and thus, more attention should be placed on primary prevention strategies.

## Figures and Tables

**Table 1:** The four definitions of MetS.

	Definition
WHO (1999) [14]	Insulin resistance is defined as type 2 diabetes mellitus (DM) or impaired fasting glucose (IFG) (>100 mg/dL) or impaired glucose tolerance (IGT), plus two of the following: Abdominal obesity (waist-to-hip ratio > 0.9 in men or >0.85 in women, or BMI > 30 kg/m^2^. Triglycerides 150 mg/dL or greater, and/or high-density lipoprotein (HDL)-cholesterol < 40 mg/dL in men and <50 mg/dL in women. Blood pressure (BP) 140/90 mmHg or greater. Microalbuminuria (urinary albumin secretion rate 20 μg/min or greater, or albumin-to-creatinine ratio 30 mg/g or greater).
IDF (2005) [1]	Central obesity (defined as waist circumference but can be assumed if BMI > 30 kg/m^2^) with ethnicity-specific values *, plus two of the following: Triglycerides 150 mg/dL or greater. HDL-cholesterol < 40 mg/dL in men and <50 mg/dL in women. BP 130/85 mmHg or greater. Fasting glucose 100 mg/dL or greater.
NCEP:ATPIII (2001) [12]	Any three or more of the following: Waist circumference > 102 cm in men, >88 cm in women. Triglycerides 150 mg/dL or greater. HDL-cholesterol < 40 mg/dL in men and <50 mg/dL in women. BP 130/85 mmHg or greater. Fasting glucose 100 mg/dL or greater.
Consensus (2009) [13]	Any three of the following: Elevated waist circumference (according to population and country-specific definitions). Triglycerides 150 mg/dL or greater. HDL-cholesterol < 40 mg/dL in men and <50 mg/dL in women. BP 130/85 mmHg or greater. Fasting glucose 100 mg/dL or greater.

* To meet the criteria, waist circumference must be as follows: for Europeans, >94 cm in men and >80 cm in women.

**Table 2:** Baseline characteristics table of the EC cohort and the control cohort.

	Control *n* = 174,097	EC *n* = 1454	*p*-Value (Mann-U)
Follow-up (years) median (IQR)	11.9 (11.2–12.6)	6.2 (3.6–9.1)	<0.001
Ethnicity *n* (%)			
White	162,146 (92.4)	1354 (93.1)	<0.001
Mixed	1371 (0.8)	7 (0.5)	<0.001
Asian/Asian British	3516 (2.0)	36 (2.4)	<0.001
Black/Black British	3274 (2.1)	17 (1.2)	<0.001
East Asian	788 (0.4)	4 (0.3)	<0.001
Other	1909 (1.1)	22 (1.5)	<0.001
NA—Not answered *n* (%)	1093 (0.6)	14 (1.0)	<0.001
Index of deprivation (England) median (IQR)	12.9 (7.4–23.3)	14.0 (8.0–23.6)	<0.001
Age at recruitment median (IQR)	55.0 (48.0–62.0)	60.0 (54.0–64.0)	<0.0001
Height (cm) mean (SD)	162.6 (6.4)	162.0 (6.2)	0.0002
Height missing *n* (%)	754 (0.4)	6 (0.4)	NS
Weight (kg) median (IQR)	68.5 (61.2–77.9)	76.7 (67.1–90.9)	<0.0001
Weight missing *n* (%)	892 (0.5)	7 (0.5)	NS
Waist circumference (cm) median (IQR)	82 (75–91)	91 (81–102)	<0.0001
Waist circumference missing *n* (%)	719 (0.4)	4 (0.3)	
BMI (kg/m^2^) median (IQR)	25.8 (23.2–29.4)	29.4 (25.4–34.9)	<0.0001
BMI missing *n* (%)	2395 (1.4)	8 (0.6)	<0.0001
Age at menarche median (IQR)	13.0 (12.0–13.0)	13.0 (12.0–14.0)	<0.0001
Pre-menopausal, *n* (%)	55,260 (33.6)	251 (18.1)	<0.0001
Post-menopause, *n* (%)	109,120 (66.4)	1134 (81.9)	<0.0001
Menopause missing *n* (%)	9717 (5.6)	69 (4.8)	<0.0001
Age at menopause median (IQR)	51.0 (48.0–53.0)	52.0 (50.0–55.0)	<0.0001
Nulliparous *n* (%)	35,745 (20.5)	365 (25.1)	<0.0001
Oral contraceptive pill (OCP) used *n* (%)	142, 452 (81.9)	1023 (70.4)	<0.0001
OCP never used *n* (%)	31,163 (17.9)	427 (29.7)	<0.0001
OCP data missing	392 (0.2)	4 (0.3)	NS
Hormone replacement therapy (HRT) used *n* (%)	50,556 (29.0)	525 (36.1)	<0.0001
HRT never used *n* (%)	123,149 (70.7)	925 (63.6)	<0.0001
HRT data missing	392 (0.2)	4 (0.3)	
Never smoked *n* (%)	106,094 (60.9)	967 (66.5)	<0.0001
Ex-smoker *n* (%)	52,105 (29.9)	410 (28.2)	<0.0001
Current smoker *n* (%)	14,929 (8.6)	68 (4.7)	<0.0001
Smoking status missing *n* (%)	969 (0.6)	9 (0.6)	NS
Diabetes mellitus (DM)—any *n* (%)	5650 (3.3)	117 (8.1)	<0.0001
Diabetes status missing	861 (0.5)	13 (0.9)	<0.0001
Taking cholesterol lowering medication *n* (%)	17,795 (10.3)	268 (18.7)	<0.0001
Anti-hypertensives *n* (%)	15,295 (8.9)	217 (15.1)	<0.0001
Polycystic ovarian syndrome *n* (%)	263 (0.2)	4 (0.3)	<0.001

**Table 3:** Frequency of MetS Components in the EC and control cohort.

	Controls *n* = 174,097	EC *n* = 1454	*p*-Value (Mann-U)
BMI ≥ 30 kg/m^2^ (%)	38,235 (22.0)	663 (45.6)	<0.001
Waist Circumference ≥ 80 cm (%)	54,148 (31.1)	810 (55.7)	<0.001
Waist-to-Hip ratio ≥ 0.85 (%)	49,993 (28.7)	609 (41.9)	<0.001
Arterial BP ≥ 130/85 mmHg (%)	47,600 (27.3)	522 (35.9)	<0.001
HbA1c ≥ 48 mmol/mol (%)	3706 (2.1)	75 (5.2)	<0.001
Fasting Glucose ≥ 100 mg/dL (%)	20,391 (11.7)	242 (16.6)	<0.001
Triglycerides ≥ 150 mg/dL (%)	46,977 (27.0)	598 (41.1)	<0.001
HDL Cholesterol ≤ 50 mg/dL (%)	57,654 (33.1)	614 (42.2)	<0.001
MetS (WHO 1998) (%)	9051 (5.2)	160 (11.0)	<0.001
MetS (NCEP:ATPIII 2001) (%)	28,916 (16.6)	500 (34.4)	<0.001
MetS IDF (2005) (%)	36,711 (21.1)	552 (38.0)	<0.001
Consensus MetS (2009) (%)	37,972 (21.8)	569 (39.1)	<0.001

**Table 4:** Different definitions of MetS and risk of EC (corrected for age, COC, HRT, age at menarche, smoking, and nulliparity).

	HR	95.0% CI for HR	*p*	SE
WHO (1998) [14]	1.928	1.633–2.276	<0.001	0.085
IDF (2005) [1]	2.080	1.869–2.314	<0.001	0.054
NCEP:ATPIII (2001) [12]	2.385	2.138–2.659	<0.001	0.056
Consensus (2009) [13]	2.091	1.880–2.325	<0.001	0.054

**Table 5:** Risk of MetS components in pre- and post-menopausal cohorts on EC development.

	Pre-Menopausal N = 249				Post Menopausal N = 1201			
	HR	95.0% CI for HR	*p*	SE	HR	95.0% CI for HR	*p*	SE
Age at menarche	0.928	0.857–1.004	0.064	0.041	0.912	0.879–0.945	<0.001	0.018
Nulliparity	1.779	1.374–2.304	<0.001	0.234	1.233	1.068–1.423	0.004	0.090
Ever used contraception	0.757	0.549–1.045	0.091	0.164	0.760	0.667–0.866	<0.001	0.066
Ever used HRT	0.866	0.673–1.115	0.266	0.129	1.006	0.894–1.132	0.922	0.060
Smoking	1.594	1.198–2.122	0.001	0.146	0.752	0.671–0.843	<0.001	0.058
Waist circumference > 80 cm	1.104	0.820–1.486	0.513	0.152	2.165	1.910–2.454	<0.001	0.064
Triglycerides > 150 mg/dL	1.534	1.182–1.991	0.001	0.133	1.228	1.089–1.386	<0.001	0.062
HDL < 50 mg/dL	1.199	0.909–1.581	0.200	0.141	1.188	1.054–1.339	0.005	0.061
BP > 130/85 mmHg	1.016	0.664–1.552	0.943	0.217	1.109	0.985–1.250	0.087	0.061
Glucose > 100 mg/dL	0.928	0.415–2.076	0.856	0.411	1.021	0.870–1.198	0.803	0.082
HbA1c > 48 mmol/mol	1.511	1.078–2.119	0.017	0.172	1.288	0.986–1.682	0.063	0.136

## Data Availability

Data can be made available upon requests made via the UK Biobank.

## Supplementary Materials

The following supporting information can be downloaded at: https://www.mdpi.com/article/10.3390/jcm14030751/s1, Table S1: Risk of MetS components in the EC and control cohort, Figure S1: Frequency histogram displaying MetS components in EC cases and controls. * denotes significant difference (*p* < 0.05). Figure S2: Frequency histogram displaying frequency of differing MetS diagnosis amongst EC cases and controls. * denotes significant difference (*p* < 0.05). Figure S3: Kaplan–Meier curves showing difference between cumulative hazard of EC development in those with MetS and those without. The differing MetS diagnostic criteria are compared. The top left compares the WHO diagnostic criteria and the top right analyses the IDF criteria. The bottom left analyses the NCEP criteria and the bottom right analyses the Consensus criteria. On the x axis, the time to EC development in years is plotted against the cumulative hazard, on the y axis.
