# Supplementary material for: The Association Between Metabolic Syndrome and the Risk of Endometrial Cancer in Pre- and Post-Menopausal Women: A UK Biobank Study
Source: J Clin Med. 2025 Jan 24;14(3):751. doi: 10.3390/jcm14030751 (PMC11818266; doi:10.3390/jcm14030751)
Supplement: Supplementary file 1 [file jcm-14-00751-s001.zip › jcm-3380964-supplementary.pdf]

Supplementary Materials

Supplementary Table S1. Risk of MetS components in the EC and control cohort

	HR	95.0% CI for HR	p	SE
Menopausal status	1.931	1.669-2.234	<0.001	0.074
Age at menarche	0.917	0.887-0.948	<0.001	0.017
Nulliparity	1.327	1.172-1.502	<0.001	0.084
Oral contraceptive pill use (ever use)	0.638	0.569-0.716	<0.001	0.059
HRT (ever use)	1.143	1.021-1.279	0.021	0.058
Smoking	0.777	0.700-0.862	<0.001	0.053
BMI 25-29 kg/m^2	1.394	1.190- 1.634	<0.001	0.112
BMI 30-34 kg/m^2	1.902	1.533- 2.360	<0.001	0.209
BMI 35-39 kg/m^2	3.564	2.793- 4.549	<0.001	0.443
BMI ≥40 kg/m^2	5.870	4.525-7.617	<0.001	0.780
Waist circumference ≥80 cm	2.212	1.977-2.474	<0.001	0.057
Triglycerides ≥150 mg/dL	1.286	1.150-1.437	<0.001	0.057
HDL ≤ 50 mg/dL	1.209	1.084-1.347	<0.001	0.055
BP ≥130/85 mmHg	1.155	1.036-1.289	0.010	0.056
Glucose ≥100 mg/dL	1.082	0.931-1.257	0.305	0.076
HbA1c ≥48 mmol/mol	1.310	1.017-1.687	0.037	0.129

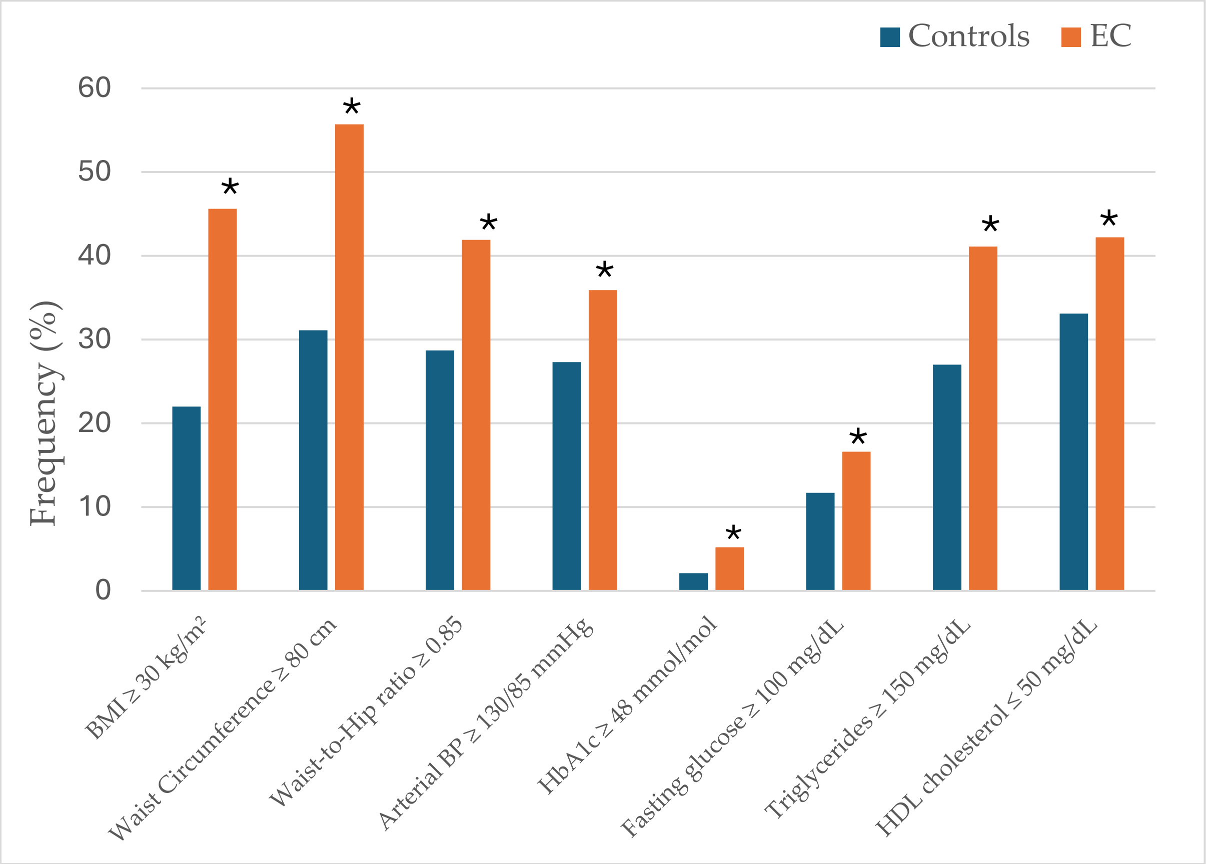

Supplementary Figure S1. Frequency histogram displaying MetS components in EC cases and controls. * denotes significant difference (P<0.05).

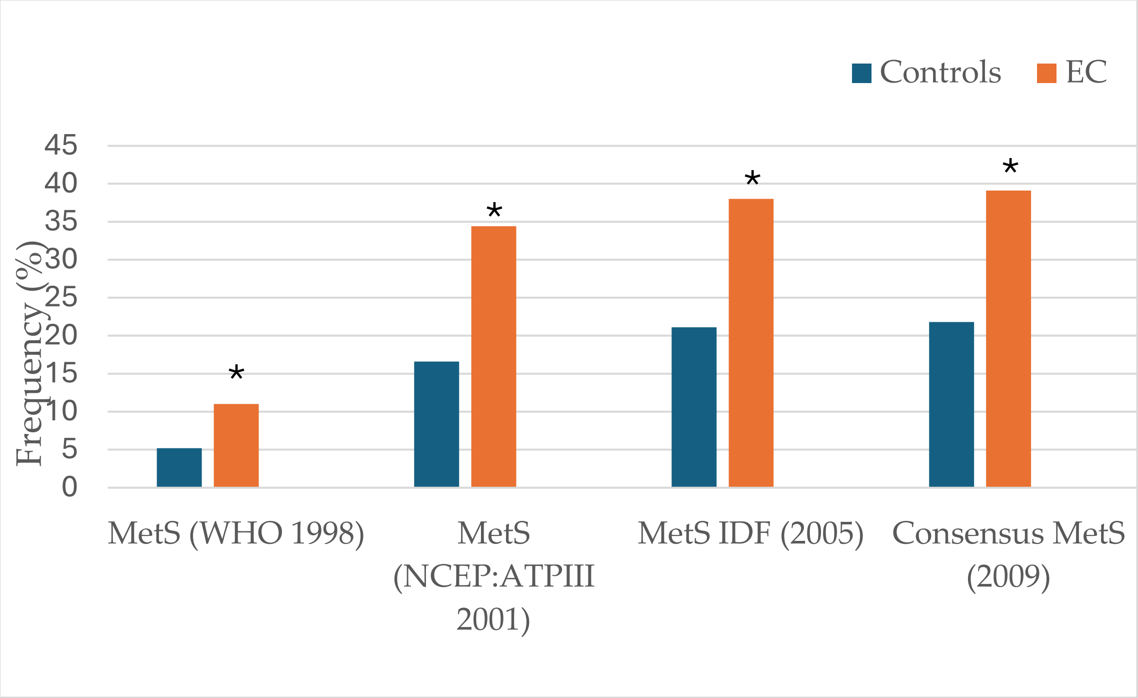

Supplementary Figure S2. Frequency histogram displaying frequency of differing MetS diagnosis amongst EC cases and controls. * denotes significant difference (P<0.05).

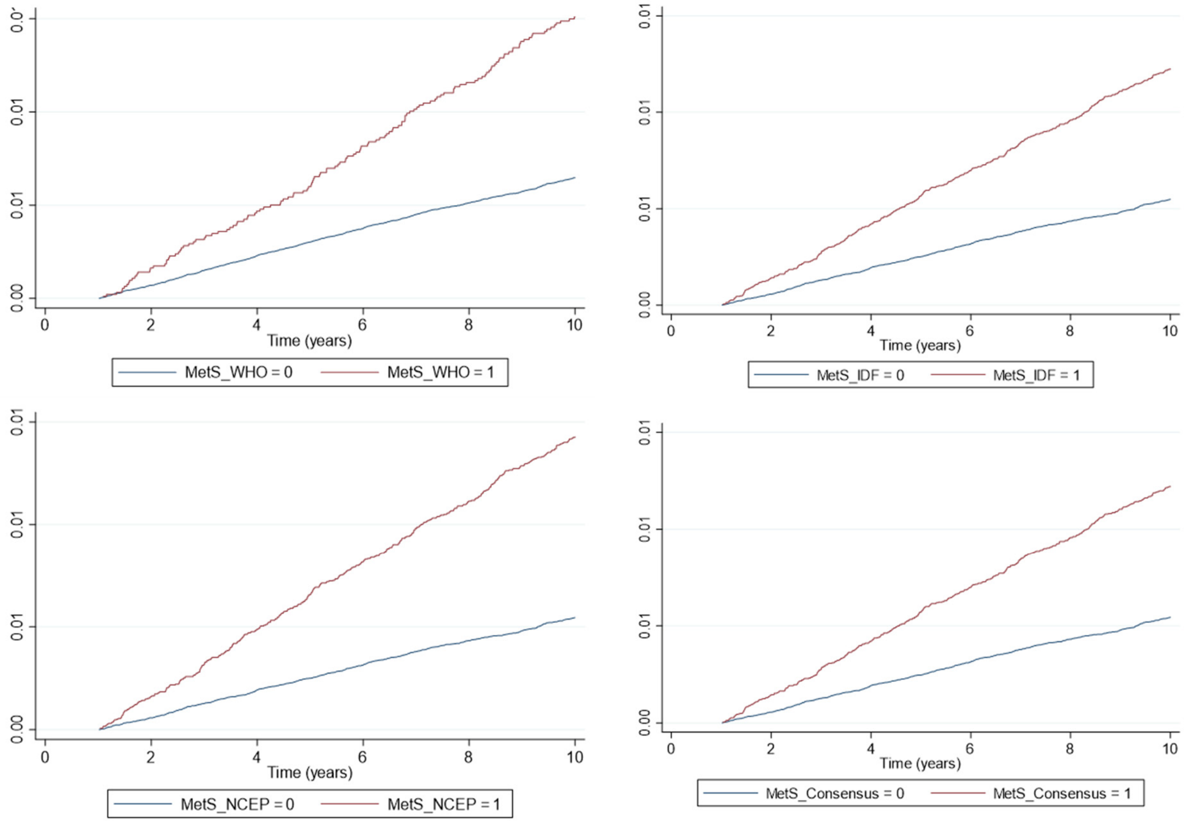

Supplementary Figure S3. Kaplan–Meier curves showing difference between cumulative hazard of EC development in those with MetS and those without. The differing MetS diagnostic criteria are compared. The top left compares the WHO diagnostic criteria and the top right

analyses the IDF criteria. The bottom left analyses the NCEP criteria and the bottom right analyses the Consensus criteria. On the x axis, the time to EC development in years is plotted against the cumulative hazard, on the y axis.
